# Gut microbiome alterations in Alzheimer’s disease

**DOI:** 10.1038/s41598-017-13601-y

**Published:** 2017-10-19

**Authors:** Nicholas M. Vogt, Robert L. Kerby, Kimberly A. Dill-McFarland, Sandra J. Harding, Andrew P. Merluzzi, Sterling C. Johnson, Cynthia M. Carlsson, Sanjay Asthana, Henrik Zetterberg, Kaj Blennow, Barbara B. Bendlin, Federico E. Rey

**Affiliations:** 1 Wisconsin Alzheimer’s Disease Research Center, University of Wisconsin School of Medicine and Public Health, 600 Highland Avenue J5/1 Mezzanine, Madison, WI 53792 USA; 2 Department of Bacteriology, University of Wisconsin-Madison, 1550 Linden Drive, Madison, WI 53706 USA; 3 Geriatric Research Education and Clinical Center, William S. Middleton Memorial Veterans Hospital, 2500 Overlook Terrace, Madison, WI 53705 USA; 4 Wisconsin Alzheimer’s Institute, University of Wisconsin School of Medicine and Public Health, WARF Building, 610 Walnut Street, 9th Floor, Suite 957, Madison, WI 53726 USA; 5 Department of Psychiatry and Neurochemistry, Institute of Neuroscience and Physiology, The Sahlgrenska Academy at the University of Gothenburg, Mölndal, Sweden; 6 Clinical Neurochemistry Laboratory, Sahlgrenska University Hospital, Mölndal, Sweden; 7 Department of Molecular Neuroscience, University College London Institute of Neurology, Queen Square, London, United Kingdom; 8 UK Dementia Research Institute at University College London, London, United Kingdom

## Abstract

Alzheimer’s disease (AD) is the most common form of dementia. However, the etiopathogenesis of this devastating disease is not fully understood. Recent studies in rodents suggest that alterations in the gut microbiome may contribute to amyloid deposition, yet the microbial communities associated with AD have not been characterized in humans. Towards this end, we characterized the bacterial taxonomic composition of fecal samples from participants with and without a diagnosis of dementia due to AD. Our analyses revealed that the gut microbiome of AD participants has decreased microbial diversity and is compositionally distinct from control age- and sex-matched individuals. We identified phylum- through genus-wide differences in bacterial abundance including decreased Firmicutes, increased Bacteroidetes, and decreased *Bifidobacterium* in the microbiome of AD participants. Furthermore, we observed correlations between levels of differentially abundant genera and cerebrospinal fluid (CSF) biomarkers of AD. These findings add AD to the growing list of diseases associated with gut microbial alterations, as well as suggest that gut bacterial communities may be a target for therapeutic intervention.

## Introduction

Despite decades of research, the etiology underlying the development of dementia due to Alzheimer’s disease (AD) remains unknown, and there are currently no preventative or disease-modifying treatments available. In the brain, AD pathology is characterized by extracellular plaques composed of amyloid-β (Aβ) peptide and intracellular neurofibrillary tangles composed of hyperphosphorylated tau protein^1^. However, what causes these hallmark features is unexplained. In recent years, researchers have proposed a potential role for pathogenic microbes, including those derived from the gut, in the development or exacerbation of AD pathology^2–5^.

Humans harbor complex communities of microbes, with the vast majority of the microbial population residing in the distal gut. Gut microbes perform key functions for human health including energy extraction, biosynthesis of vitamins, protection against pathogen overgrowth, and education of the immune system^6^. Microbial colonization of the gut occurs during birth, is highly dynamic through infancy, and resembles adult structure by about 3 years of age^7^. Thereafter, the composition of the microbiome within an individual remains generally stable^8^, albeit with substantial interpersonal variation, particularly in elderly individuals^9^.

Alterations in the composition of this complex ecosystem have been associated with the development of a variety of gastrointestinal and metabolic diseases including inflammatory bowel disease (IBD), obesity, diabetes, and insulin resistance^10^. More recently, the influence of gut microbiota on central nervous system function – often referred to as the gut-brain axis – has received significant attention, and alterations in the gut microbiome have been associated with neurological conditions including autism spectrum disorder, multiple sclerosis, and Parkinson’s disease^11–13^.

With respect to dementia, a recent study in cognitively impaired elderly participants investigated a limited number of pro- and anti-inflammatory gut bacterial taxa and found altered abundance in individuals with positive amyloid positron emission tomography (PET) imaging^14^. In addition, recent studies in transgenic mouse models of AD have demonstrated that manipulating gut microbiota can influence cerebral amyloid deposition^15,16^. However, to date there have been no comprehensive surveys of whole gut microbiota in humans with AD. In this study, we performed bacterial 16S ribosomal RNA (rRNA) gene sequencing of DNA isolated from fecal samples in order to characterize the gut microbial communities in individuals with and without a clinical diagnosis of dementia due to AD. In addition, we examined the relationship between gut microbiota and AD pathology as measured by cerebrospinal fluid (CSF) biomarkers of AD.

## Results

### Study Design and Participant Characteristics

Participants were recruited from the Wisconsin Alzheimer’s Disease Research Center (ADRC) and the Wisconsin Registry for Alzheimer’s Prevention (WRAP) study (see Methods). Gut microbiome compositional analysis was performed on fecal samples collected from home-dwelling participants with dementia due to AD (n = 25), and age- and sex-matched Control participants (n = 25). Table 1 reports participant characteristics. AD and Control groups did not differ with respect to age, sex, ethnicity, BMI, or diabetes status. There was no difference between groups in total score on a 15-item food questionnaire based on the Mediterranean-DASH Intervention for Neurodegenerative Delay (MIND) Diet^17^, which provided a semi-quantitative measure of dietary intake and allowed us to asses dietary differences. As expected, the *APOE* ε4 genotype was more prevalent in the AD group. The majority of AD participants had very mild or mild dementia, with clinical dementia rating (CDR) scores ranging from 0.5–2. Medication information is reported in Supplementary Table S1. The AD group had a greater number of participants taking selective serotonin reuptake inhibitors (SSRIs), and all but one AD participant was taking an acetylcholinesterase inhibitor (donepezil or rivastigmine) and/or memantine.

**Table 1:** Participant characteristics.

	**Control**	**AD**	***p*** **value**
n	25	25	
Age (yrs, mean ± SD)	69.3 ± 7.5	71.3 ± 7.3	0.346
Sex (% Female)	72% (18/25)	68% (17/25)	0.785
Clinical dementia rating (CDR) score			*NA*
0–normal	100% (25/25)	0	
0.5–very mild dementia		40% (10/25)	
1–mild dementia		36% (9/25)	
2–moderate dementia		24% (6/25)	
*APOE* ε4 genotype	20% (5/25)	72% (18/25)	<0.001*
Ethnicity (% Caucasian)	96% (24/25)	92% (23/25)	0.552
BMI (kg/m^2^, median [IQR])	26.1 [24.3–33.2]	26.0 [22.9–29.1]	0.467
Diabetes diagnosis	2/25	2/25	1.000
MIND Diet total score (mean ± SD)	7.6 ± 2.2	6.6 ± 2.7	0.160
Bristol stool scale score (mean ± SD)	3.5 ± 1.7	3.8 ± 1.2	0.561

### Composition of the Gut Microbiome of Control and AD Groups

Sequencing of the V4 region of the 16S rRNA gene generated a total of 4.8 million sequence reads (mean ± SD: ~96,000 ± 32,000 reads/participant), which were clustered into operational taxonomic units (OTUs) at 97% similarity and assigned taxonomic classifications down to the lowest phylogenetic level possible (see Methods). The final OTU dataset for AD and Control groups consisted of 972 OTUs classified to 95 genera, 46 families, 24 orders, 17 classes, and 9 phyla. Between groups, there were no differences in percentages of sequences classified to the phylum (Control: 99.6 ± 0.5%, AD: 99.6 ± 0.9%) or genus level (Control: 79.6 ± 7.4%, AD: 83.1 ± 8.1%). Across all 50 participants, the dominant phyla were Firmicutes and Bacteroidetes, which respectively made up 78% (78.1 ± 8.7%) and 15% (14.9 ± 8.4%) of total abundance, with lower contributions from Actinobacteria (2.6%), Verrucomicrobia (2.6%), and Proteobacteria (1.1%) (Supplementary Fig. S1). The predominant bacterial families for all participants were *Lachnospiraceae* (39.1%), *Ruminococcaceae* (29.6%), and *Bacteroidaceae* (9.8%), followed by *Verrucomicrobiaceae* (2.6%)*, Clostridiales* (1.9%), and *Bifidobacteriaceae* (1.5%) (Supplementary Fig. S1).

### AD is Associated with Changes in the Gut Microbiome

**Figure 1:**
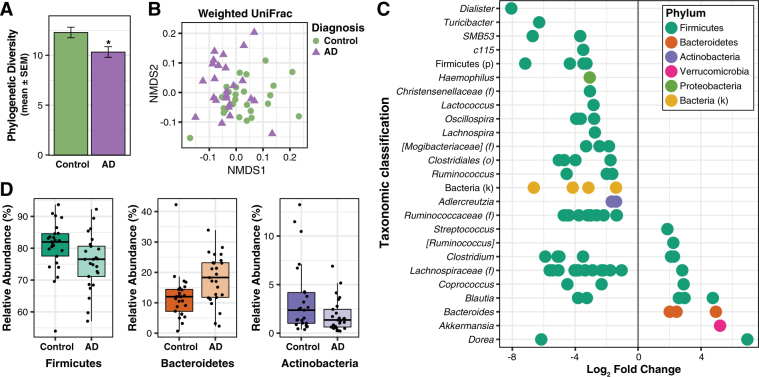
Alzheimer’s disease is associated with alterations in gut microbiome composition. (**A**) Faith’s Phylogenetic Diversity is decreased in the microbiome of AD participants. *p < 0.05. (**B**) Non-metric multidimensional scaling (NMDS) plot of weighted UniFrac analysis of relative sample OTU composition. NMDS analysis was limited to two dimensions, with a stress measurement of 0.17. Each dot represents a scaled measure of the composition of a given participant, color- and shape-coded by cohort. (**C**) Differential abundance analysis identified 14 OTUs that were increased and 68 OTUs that were decreased in AD relative to Control participants (*p* < 0.05, FDR-corrected). Each point represents an OTU. Data plotted as log_2_ fold change; OTUs to the right of the zero line are more abundant and OTUs to the left of the zero line are less abundant in AD compared to Control groups. OTUs are organized on the y-axis according to the lowest taxonomic classification possible. (**D**) OTUs grouped at the phylum level and analyzed using *Metastats* show that AD participants have decreased abundance of Firmicutes and Actinobacteria, and increased abundance of Bacteroidetes compared to Control participants (*p* < 0.05, FDR-corrected). Tukey plots show median, IQR, and participant data points for phylum relative abundance.

The composition of the gut microbiome was characterized using traditional ecological measures including richness (the number of unique OTUs present in a participant), alpha diversity (the richness and abundance of OTUs within each participant), and beta diversity (the similarity or difference in composition between participants). For microbiome richness estimates, we used the Abundance-based coverage estimator (ACE) and Chao1; these metrics use non-parametric modeling to calculate a conservative estimate of total OTU richness for each participant. The microbiome of AD participants had reduced richness, with both ACE and Chao1 significantly decreased in the AD group compared to the Control group (*t*-test; ACE: DF = 48, *t* = 3.05, *p* = 0.004; Chao1: DF = 48, *t* = 2.98, *p* = 0.004) (Supplementary Fig. S2). For alpha diversity metrics, we used the Inverse Simpson and Shannon Indexes, and Faith’s Phylogenetic Diversity (PD), an alpha diversity metric that also incorporates phylogenetic relationships. While there was a trend towards a decrease in the Inverse Simpson Index between Control and AD groups (Mann-Whitney; *U* = 227.0, *p* = 0.097), both the Shannon Index and Faith’s PD were significantly decreased in AD participants compared to Control participants (*t*-test; Shannon: DF = 48, *t* = 2.44, *p* = 0.019, PD: DF = 48, *t* = 2.59, *p* = 0.013) (Fig. 1A, Supplementary Fig. S2). With respect to beta diversity, Bray-Curtis dissimilarity and UniFrac analysis (weighted and unweighted) demonstrated compositional differences in the microbiome between AD and Control groups (PERMANOVA, Bray-Curtis: *F* = 2.87, *p* < 0.001, weighted UniFrac: *F* = 3.84, *p* < 0.001; unweighted UniFrac: *F* = 2.60, *p* < 0.005) (Fig. 1B, Supplementary Fig. S3).

Differential abundance analysis of taxa at the OTU level revealed that the microbiome of AD participants showed significantly altered abundance of 82 OTUs relative to the Control group, with 14 OTUs more abundant and 68 OTUs less abundant in AD (Fig. 1C, Supplementary Table S2). Plotting these 82 OTUs as a phylogenetic tree-ordered and diagnosis-grouped heat map shows clear differences in OTU abundance distributions between Control and AD groups (Supplementary Fig. S4).

**Figure 2:**
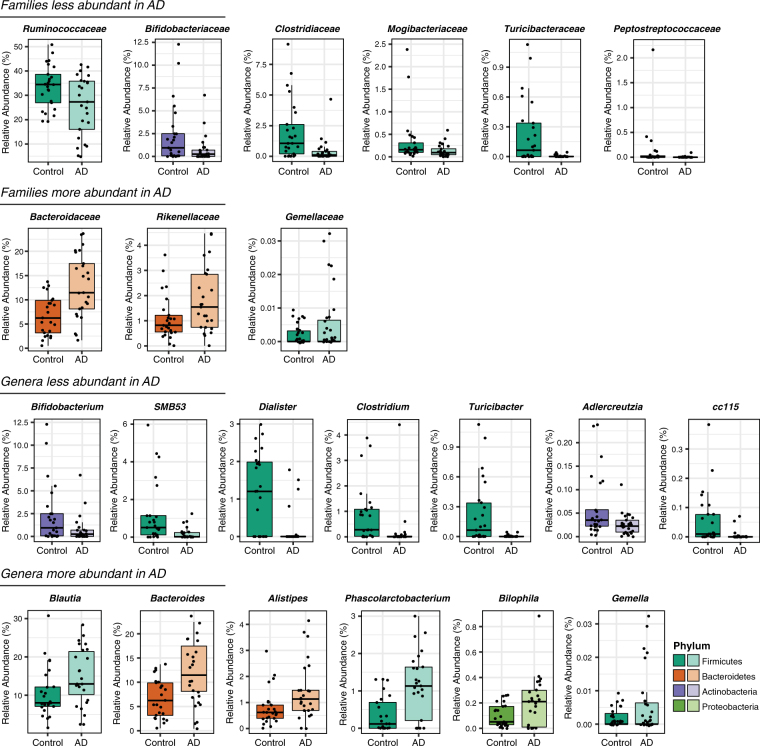
Bacterial families and genera differentially represented in feces from AD participants compared to Control participants (*p* < 0.05, FDR-corrected). Tukey plots are colored by phylum and show median, IQR, and participant data points for genus or family relative abundance.

OTUs were taxonomically grouped and differential abundance was analyzed at the phylum, family, and genus levels using *Metastats* (Supplementary Table S3). At the phylum level, AD participants had decreased abundance of Firmicutes and Actinobacteria, and increased abundance of Bacteroidetes compared to Control participants (Fig. 1D). Within Firmicutes, the families *Ruminococcaceae*, *Turicibacteraceae*, *Peptostreptococcaceae*, *Clostridiaceae*, and *Mogibacteriaceae*, and the genera *SMB53* (family *Clostridiaceae)*, *Dialister*, *Clostridium*, *Turicibacter*, and *cc115* (family *Erysipelotrichaceae*) were all less abundant in AD participants, while the family *Gemellaceae* and the genera *Blautia*, *Phascolarctobacterium*, and *Gemella* were more abundant in AD participants (Fig. 2). Within Bacteroidetes, *Bacteroidaceae* and *Rikenellaceae* at the family level, and *Bacteroides* and *Alistipes* at the genus level were more abundant in AD participants. The decrease in Actinobacteria was reflected by decreased *Bifidobacteriaceae* at the family level and by decreased *Bifidobacterium* and *Adlercreutzia* at the genus level. Additionally, the genus *Bilophila* in the phylum *Proteobacteria* was more abundant in AD participants.

Predictive metagenomics analysis (PICRUSt^18^) identified potential functional changes in the gut microbiome of AD participants. These include increases in predicted gene content in KEGG pathways related to metabolism and biosynthesis, including oxidative phosphorylation, carbohydrate metabolism, and amino acid metabolism (Supplementary Fig. S5), and decreases in predicted gene content in KEGG pathways related to signal transduction and cell motility, including bacterial chemotaxis pathways, secretion systems, bacterial motility proteins, and two-component signal transduction systems.

### Differentially Abundant Genera Are Correlated with CSF Biomarkers of AD Pathology

Our primary microbiome compositional analysis identified 13 genera as differentially abundant between AD and Control groups. We next examined the relationship between the relative abundance of these 13 taxa and levels of CSF biomarkers in a subset of participants who had also undergone lumbar puncture. This analysis included microbiome and CSF data from 9 AD group participants, and 31 non-demented (ND) participants (10 from the Control group, and an additional 21 largely younger participants not selected in the original random matching with AD participants). Correlations were calculated using data from the total group of 40 participants, as well as separately for ND participants and AD participants. CSF biomarkers included Aβ_42_/Aβ_40_, phosphorylated tau (p-tau), the ratio of p-tau/Aβ_42_, and chitinase-3-like protein 1 (YKL-40). CSF Aβ_42_/Aβ_40_ is an indicator of amyloid burden, with lower levels in the CSF reflecting greater amyloid deposition in the brain; p-tau is a marker of neurofibrillary tangles, with higher levels reflecting greater tangle pathology in the brain; the ratio of p-tau/Aβ_42_ incorporates both aspects of pathology, with higher values implying greater AD pathology^19^. YKL-40 is a marker of astroglial and/or microglial activation, and has been shown to be elevated in CSF of individuals with dementia due to AD^20–22^. Supplementary Table S4 reports participant characteristics, CSF biomarker levels, and relative abundances of genera.

**Figure 3:**
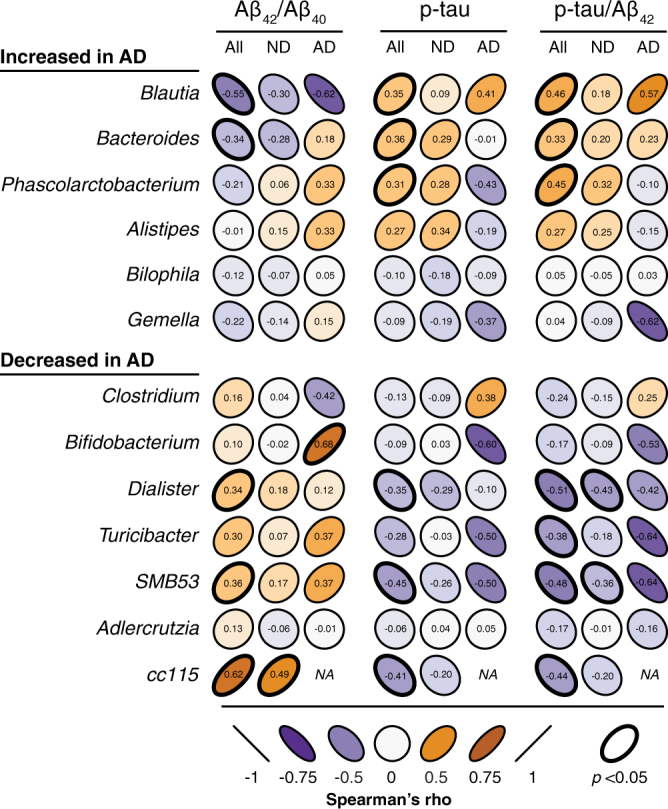
Bacterial taxa correlate with CSF biomarkers of AD pathology. 13 genera identified as differentially abundant in AD were correlated with CSF biomarkers of AD including the Aβ_42_/Aβ_40_ ratio (with lower CSF levels reflecting greater amyloid deposition in the brain), phosphorylated tau (p-tau), and the p-tau/Aβ_42_ ratio (which incorporates both facets of AD pathology). Correlations were calculated separately for all 40 participants (All), 31 non-demented participants (ND), and 9 AD participants (AD). In general, genera identified as more abundant in AD were associated with greater AD pathology, while genera identified as less abundant in AD were associated with less AD pathology. Genera are ordered from most to least abundant. Shape and color of ellipses represent strength of Spearman’s rank correlation coefficients (rho). Bolded ellipse borders represent significant correlations (two-sided, *p* < 0.05 uncorrected).

Across all 40 participants included in this analysis, we observed generally consistent trends between bacterial relative abundance and CSF biomarkers of AD pathology (Fig. 3). The direction of these trends was largely similar for both AD participants and healthy non-demented participants. For genera that are more abundant in AD, we observed a relationship between increased bacterial abundance and greater AD pathology, which was indicated by predominantly negative correlations between bacterial abundance and CSF Aβ_42_/Aβ_40_ (suggesting greater abundance is associated with greater amyloid burden in the brain), and predominantly positive correlations between bacterial abundance and CSF p-tau and p-tau/Aβ_42_. These relationships were especially strong in those genera with overall greater relative abundance, particularly in *Bacteroides* and *Blautia*. Similarly, for those genera that are less abundant in AD, we observed a relationship between decreased bacterial abundance and greater AD pathology, with levels of *SMB53* and *Dialister* showing the strongest correlations with CSF AD biomarkers. Notably, in both more and less abundant genera, the strongest correlations between bacterial abundance and CSF AD biomarkers were in the same direction whether including only AD participants, only healthy non-demented participants, or all participants. Additionally, in AD participants, we observed a relationship between increased abundance of *Bacteroides* and increased CSF YKL-40 levels, and a relationship between decreased abundance of both *Turicibacter* and *SMB53* and increased CSF YKL-40 levels (Supplementary Fig. S6).

## Discussion

Despite the proposed role of gut microbiota in the development or progression of AD^4,5,23^, there have been no comprehensive surveys of the gut microbiome in individuals with AD. In this study, we performed bacterial 16S rRNA gene sequencing on DNA isolated from fecal samples in order to compare the composition of the gut microbiome in participants with and without a diagnosis of dementia due to AD. We discovered that the gut microbiome of AD participants has decreased microbial richness and diversity and a distinct composition compared to asymptomatic age- and sex-matched Control participants. We also identified several broad taxonomic differences between AD and Control groups, and determined that levels of differentially abundant genera correlate with CSF biomarkers of AD pathology.

The decreased richness and diversity in our study broadly parallels results observed in other conditions linked to gut microbiome alterations, including obesity, diabetes, IBD, and Parkinson’s disease^13,24–26^. Furthermore, PICRUst analysis revealed broad functional changes in predicted metabolism, bacterial cell motility, and signal transduction pathways in the gut microbiome of AD participants. While the specific bacteria responsible for these compositional and functional alterations may differ between conditions, it has been proposed that these broad-scale changes in gut microbiota (often referred to as “dysbiosis”) may play important roles in disease progression and maintenance, potentially through immune activation and systemic inflammation^27^. While it is unclear how the gut influences the development of neuropathology, substantial evidence supports the existence of a gut-brain axis that allows bi-directional communication between the gut and brain through several pathways including neural, endocrine, and immune mechanisms^11,28^. Within this framework, alterations in gut microbial communities in patients with AD may result in pathophysiological changes in the brain. In support of this hypothesis, a recent study showed that transgenic AD mice raised under germ-free conditions have less cerebral amyloid deposition than conventionally-raised AD mice, indicating that gut microbiota influence the development of amyloid pathology^16^.

In our study, the phylum Firmicutes as a whole, as well as several families, genera, and 61 OTUs classified within Firmicutes were decreased in the AD group. A reduction in Firmicutes has been reported in the microbiome of individuals with type 2 diabetes^25^ as well as obesity^29^ (although others have reported increased Firmicutes in obesity^24,30^). Notably, diabetes and insulin resistance increase the risk of developing AD^31–33^. We have recently reported that insulin resistance is associated with decreased cerebral glucose metabolism and increased amyloid deposition in asymptomatic middle-aged adults enriched for risk of AD^34,35^. Thus, a potential mechanism by which microbial alterations in the gut may influence AD pathology is through promoting the development of insulin resistance and diabetes. While AD and Control groups did not differ with respect to diabetes prevalence (Table 1), sub-clinical differences in insulin or glucose metabolism cannot be ruled out. Further investigation will be needed to explore the relationship between microbiota and insulin resistance in AD.

In participants with AD, we observed an increase in the phylum Bacteroidetes, which was reflected by increased *Bacteroidaceae* at the family level, and increased *Bacteroides* at the OTU and genus level. The phylum Bacteroidetes encompasses a diverse and abundant group of gram-negative commensal bacteria in the gut^36^, including the genus *Bacteroides*, which has been detected at higher levels in the gut of individuals with type 2 diabetes^25^ and in patients with Parkinson’s disease^13^, a neurodegenerative disorder. The major outer membrane component of gram-negative bacteria is lipopolysaccharide (LPS), which is capable of triggering systemic inflammation and the release of pro-inflammatory cytokines after translocation from the gut to systemic circulation^37^. Additionally, *in vitro* and *in vivo* studies have demonstrated an association between bacterial endotoxins (e.g. LPS) and AD pathology. Co-incubation of Aβ peptide with LPS potentiates amyloid fibrillogenesis^38^, and systemic injection of LPS in wild-type and transgenic AD mice results in greater amyloid deposition and tau pathology^39–42^. In humans, intestinal permeability increases with age^43^, and elderly individuals show an association between increased LPS-binding protein (a marker of microbial translocation) and inflammation^44^. Moreover, a recent study involving postmortem brain tissue from patients with AD showed that LPS and gram-negative *E*. *coli* fragments co-localize with amyloid plaques^45^. Thus, increased abundance of gram-negative intestinal bacteria such as *Bacteroides* in participants with AD may result in increased translocation of LPS from the gut to systemic circulation, which in turn may contribute to or exacerbate AD pathology through inflammation or other mechanisms.

Additionally, compared to control participants, AD participants in our study exhibited decreased Actinobacteria. These differences were mostly driven by changes in *Bifidobacterium*. Actinobacteria, particularly members of the *Bifidobacterium* genus, are an important bacterial inhabitant of the human gut across the lifespan, and their beneficial health effects have been well-documented^46,47^. In particular, certain species of *Bifidobacterium* are associated with anti-inflammatory properties and decreased intestinal permeability^48^. Additionally, supplementation with *Bifidobacterium* has been shown to decrease LPS levels in the intestine and improve gut mucosal barrier properties in mice^49,50^. Interestingly, in germ-free mice colonized with human gut microbiota, increased levels of *Bifidobacterium* are associated with decreased bacterial translocation to systemic circulation, while increased levels of *Bacteroides* have been shown to increase bacterial translocation^51^. Considering our present findings, increased *Bacteroides* and decreased *Bifidobacterium* in AD participants may represent a gut microbial phenotype with particular propensity for translocation of pro-inflammatory bacterial components. Furthermore, several *Bifidobacterium* species are widely used as probiotics. A small study of probiotics that included *Bifidobacterium* demonstrated a change in Mini-Mental State Examination scores after a 12-week intervention among participants with severe dementia^52^. Taken together with the decreased abundance of *Bifidobacterium* in AD participants observed in our study, larger trials may be warranted, particularly in earlier disease stages.

Finally, we observed correlations between levels of differentially abundant gut microbiota and CSF biomarkers of AD pathology in a subset of participants that had also undergone lumbar puncture. In general, genera identified as more abundant in AD were associated with greater AD pathology while genera identified as less abundant in AD were associated with less AD pathology. These effects were most prominent when examining CSF p-tau/Aβ_42_, a composite measure of AD pathology. Interestingly, even among non-demented participants who had undergone lumbar puncture, we found a relationship between genera that were either more or less abundant in AD and markers of amyloid and tau protein, even in the absence of dementia. In particular, *Dialister* and *SMB53* showed the strongest correlations in non-demented participants, with greater abundance of these bacteria associated with less AD pathology, suggesting these bacterial taxa may be protective against development or progression of AD pathology. We also observed significant associations in AD participants between CSF YKL-40 and abundance of *Bacteroides*, *Turicibacter*, and *SMB53* (family *Clostridiaceae*). While these findings support a link between altered gut bacterial abundance and glial activation in AD, this relationship is less clear in healthy non-demented individuals and requires further investigation.

A limited number of studies have attempted to address the role of gut microbiota in AD. A recent investigation in cognitively-impaired older adults (without an AD diagnosis) reported increased abundance of the pro-inflammatory bacteria *Escherichia*/*Shigella* and decreased abundance of the anti-inflammatory bacteria *Eubacterium rectale* in individuals with evidence of amyloid deposition on PET imaging compared to individuals who were amyloid negative^14^. While those results support a link between gut microbiota and brain amyloidosis, the study only investigated the abundance of six pre-selected bacterial taxa using quantitative PCR rather than the broader approach used here. Additionally, in a recent AD mouse microbiome study using 16S rRNA sequencing, *APP/PS1* transgenic mice showed increased *Helicobacteraceae* and *Desulfovibrionaceae* at the family level, increased *Odoribacter* and *Helicobacter*, and decreased *Prevotella* compared to wild-type mice^53^. However, while the anatomy and physiology of the gastrointestinal tract of humans and mice share many characteristics^54^, there are also substantial differences with respect to resident bacterial communities^30^, which makes comparing taxa and changes in abundance between these studies difficult.

While AD participants were well-matched to our Control participants (suggesting that the gut microbiome differences we observed were not likely the result of age, sex, BMI, or dietary differences between groups), they did differ with respect to the use of selective serotonin reuptake inhibitors (SSRIs) and AD medications. We did not find differences in microbial richness, diversity, or relative abundance of the 13 genera identified as altered in AD between AD participants taking SSRIs and AD participants not taking SSRIs (Supplementary Table S5), suggesting that these medications are not influencing our results. Nearly all AD participants in our study were taking the AD medications donepezil or rivastigmine (acetylcholinesterase inhibitors), and/or memantine (an NMDA receptor antagonist). It is unknown how these medications affect the gut microbiome. The most common side effects reported for acetylcholine esterase inhibitors are gastrointestinal upset^55^, both nausea and diarrhea, which could influence microbiota composition. It is worth noting that our participants did not report chronic constipation or diarrhea, and there was no difference between groups on the Bristol stool scale (Table 1), which can be used as a surrogate for stool transit time. Still, we recognize that we cannot completely rule out the effect of AD medication use on our results. Further work, including animal experiments and longitudinal human studies, will be needed to determine the cause-effect relationship between gut microbiota and pathogenesis of AD. Determining the role of gut bacteria in the progression or maintenance of AD may lead to novel interventional approaches that alter or restore healthy gut bacterial composition, or identification of microbial metabolites that are protective against AD.

## Methods

### Participants

Participants with dementia due to AD (n = 25) were recruited from the Wisconsin Alzheimer’s Disease Research Center (ADRC). Non-demented participants (n = 94) were recruited from both the ADRC and the Wisconsin Registry for Alzheimer’s Prevention (WRAP) study^56^. The University of Wisconsin Health Science Institutional Review Board approved all study procedures, and all experiments were performed in accordance with relevant guidelines and regulations. All participants provided written informed consent to be involved in this study.

Exclusion criteria for this study included any significant neurologic disease, history of alcohol/substance dependence, major psychiatric disorders (including major depression), or any other significant medical illness. Microbiome-specific exclusion criteria included: the use of systemic antibiotics in the previous 6 months prior to providing the fecal sample; corticosteroid use (oral, IV, nasal, or inhaled); immune stimulating medications; immunosuppressive agents; large doses of commercial probiotics consumed (greater than or equal to 10^8^ cfu or organisms per day); major dietary change during previous month (defined as eliminating or significantly increasing a major food group); major GI tract surgery in past 5 years (with the exception of cholecystectomy and appendectomy); major bowel resection at any time; active uncontrolled GI disorders or diseases including inflammatory bowel disease (IBD), indeterminate colitis, irritable bowel syndrome (IBS), persistent, infectious gastroenteritis, colitis or gastritis, persistent or chronic diarrhea of unknown etiology, *Clostridium difficile* infection (recurrent) or *Helicobacter pylori* infection (untreated), or chronic constipation.

At the time of fecal sample collection, participants completed a short questionnaire regarding recent antibiotic or probiotic use, as well as current and past gastrointestinal/metabolic conditions. Additionally, participants also completed a 15-item self-report diet questionnaire developed by Martha Clare Morris (Rush University) and based on the MIND Diet^17^, which allowed us to assess dietary differences between groups. We also used information/data collected from participants at annual and bi-annual ADRC and WRAP study visits including medication use, medical conditions/diagnoses, clinical dementia rating (CDR) scores, and CSF biomarker data (see below). Body mass index (BMI) was calculated using the height and weight of each participant at their most recent ADRC or WRAP study visit. *APOE* ε4 genotyping procedures have been described previously^57^, and participants were categorized as non-carriers (zero ε4 alleles) or *APOE* ε4 carriers (one or two ε4 alleles). Participants with AD were diagnosed using the NINDS/ADRDA criteria^58^, and confirmed by a multidisciplinary consensus diagnostic panel.

Of the 119 participants recruited, six non-demented participants were excluded from analyses due to antibiotic or probiotic use at the time of fecal sample collection that was not reported during initial screening. Participants recruited from the WRAP study were largely younger than those recruited from the ADRC, thus for our primary compositional analysis we age- and sex-matched the 25 AD participants 1-to-1 from the remaining 88 asymptomatic control participants using case-control matching in SPSS with an age tolerance of 4.5 years to create an equal-sized Control group. For secondary CSF correlational analysis, we used microbiome and CSF data from 9 AD cohort participants, and 31 non-demented (ND) participants, including 10 from the age- and sex-matched Control cohort, and an additional 21 largely younger participants not included in the primary analysis.

### Fecal sample collection and bacterial 16S rRNA sequencing and processing

All participants involved in the study resided at home, where fecal sample collection occurred. Participants returned by overnight delivery sample collection kits, packaged within insulated containers and chilled with frozen gel packs; all samples included in this study arrived chilled and 92% were processed and frozen (see below) the day following home collection by an individual who was blind to the participant’s cohort/diagnosis. Upon receipt, chilled samples were weighed, scored on the Bristol stool scale^59^, subsampled (~100 mg) into prepared sterile bead beating tubes, and stored at −80 °C until processing.

Fecal samples, suspended in lysis buffer/phenol:chloroform, were processed by bead beating^24^ and the genomic DNA in the recovered aqueous phase then precipitated with the addition of 0.1-volume 3 M sodium acetate and 1-volume isopropanol, incubated on ice, and centrifuged (4 °C, 20 min at 18,000 x *g*). After rinsing with 100% ethanol and drying, the DNA pellet was dissolved in TE buffer (10 mM Tris-HCl pH 8.0, 1 mM EDTA) then column-purified using the NucleoSpin Gel and PCR Clean-up kit (Macherey-Nagel Inc., Bethlehem, PA). DNA concentration was measured using the Qubit BR dsDNA assay (Invitrogen, Eugene, OR). The variable region V4 amplicon of the bacterial 16S rRNA gene was amplified in duplicate reactions/sample (plus a no-template control for each primer set) using 8-bp barcoded forward and reverse primers (0.4 μM)^60^, 12.5 ng template, and KAPA HiFi HotStart DNA polymerase (KAPA Biosystems, Wilmington, MA). Reactions were agarose gel-checked and duplicates combined, purified (NucleoSpin columns), and the DNA quantified (Qubit). The final equimolar pool was sequenced on the Illumina MiSeq platform (paired end, 2 × 250-bp).

Sequence processing and cleanup was performed using mothur v1.39.1^61^ and a previously described prototcol^60^. Briefly, quality-filtered, paired-end duplex sequence reads were combined into contigs, and sequences with ambiguous base pairings, sequences longer that 275-bp, and homopolymers greater than 8-bp were removed. Sequences were then aligned to the SILVA 16S rRNA gene reference alignment database, and chimeric sequences were identified and removed. Finally, remaining sequences with 97% similarity were clustered into operational taxonomic units (OTUs) using the OptiClust algorithm^62^ and assigned the lowest possible taxonomic classifications from the GreenGenes reference database (v13.8) using a naive Bayesian classifier requiring an 80% confidence score. As it has been demonstrated that quality-filtering 16S amplicon sequence reads can greatly improve accuracy of microbial community analysis^63^, OTUs with <0.001% of total sequence reads were filtered out from the dataset to account for sequencing errors.

### Microbial community composition and differential abundance statistical analysis

Richness (ACE, Chao1) and alpha diversity (Inverse Simpson, Shannon Index) metrics were calculated at the OTU-level in mothur by performing 1000 iterations of random subsampling to 31,396 reads (the lowest single participant number of sequences) from each participant. Faith’s Phylogenetic Diversity (PD) was calculated from a neighbor-joined phylogenetic tree created in R v3.3.2 using normalized OTU-level data and the *vegan*, *phyloseq*, and *ape* packages. Beta diversity metrics were computed using normalized OTU-level data in R and included Bray-Curtis dissimilarity, and weighted and unweighted UniFrac. To detect differences in richness and alpha diversity between groups, we used independent two-sample t-tests for normally distributed measures or Mann-Whitney *U* tests for non-normally distributed measures in SPSS. To detect statistical differences in beta diversity metrics between groups, we used permutational multivariate analysis of variance (PERMANOVA) in the *vegan* package in R.

Differential abundance of taxa between AD and Control groups was determined at the OTU level using the *DESeq2* package in R. *DESeq2* is a statistical method developed to detect differential expression in RNA-seq count data while accounting for library size differences and biological variability^64^. It has recently been demonstrated that applying these methods to microbiome OTU count data leads to improvements in detecting differential abundance compared to simple proportions or rarefying^65^. Results were expressed as log_2_ fold change in AD participants relative to Control participants. Relative abundance comparisons at the genus, family, and phylum levels were performed on normalized data in mothur using 10,000 iterations of *Metastats*, a statistical method employing non-parametric t-tests, Fisher’s exact tests, and false discovery rate (FDR) correction to detect differentially abundant features^66^. OTUs present in less than 20 participants and those OTUs that could not be classified down to the desired level were excluded from the *Metastats* analysis.

### PICRUSt predictive functional metagenomics analysis

We used PICRUSt^18^ to detect predicted functional differences in microbial communities between AD and Control participants. Briefly, OTUs were re-assigned PICRUSt-compatible taxonomic classifications using the GreenGenes version 13.5 reference database, and then normalized by 16S rRNA copy number. The resulting normalized OTU table was then used for prediction of KEGG orthologs (KOs) based on bacterial composition. The weighted nearest sequenced taxon index (NSTI), a quality metric of the phylogenetic distance between the input OTUs of our samples and the reference OTUs used for metagenomic prediction, was 0.07 ± 0.01 (mean ± SD) for our samples. KOs were collapsed into hierarchical KEGG pathways using the categorize by function command in PICRUSt, and the linear discriminate analysis (LDA) effect size (LEfSe)^67^ implementation in mothur was used to detect differences in level 2 and level 3 KEGG pathways. LDA scores (log 10) of significantly different pathways were plotted as bars using *ggplot2* in R.

### CSF collection and microbiome correlation statistical analysis

CSF was collected via lumbar puncture in the morning after a 12hr fast with a Sprotte 25-or 24-gauge spinal needle at the L3/4 or L4/5 interspace using gentle extraction into propylene syringes. CSF (~22 mL) was then combined, gently mixed and centrifuged at 2,000 x *g* for 10 minutes. Supernatants were frozen in 0.5 mL aliquots in polypropylene tubes and stored at −80 °C.

CSF measures included the Aβ_42_/Aβ_40_ ratio, phosphorylated tau (p-tau), and the p-tau/Aβ_42_ ratio. Using the Aβ_42_/Aβ_40_ ratio normalizes CSF Aβ_42_ for the total amount of Aβ peptides that are present in CSF and shows better correspondence with brain amyloid deposition as well as superior diagnostic performance than CSF Aβ_42_ alone^68^. For the Aβ_42_/Aβ_40_ ratio, CSF Aβ_42_ and CSF Aβ_40_ were quantified separately by electrochemiluminescence (ECL) using an Aβ triplex assay (MSD Human Aβ peptide Ultra-Sensitive Kit, Meso Scale Discovery, Gaithersburg, MD). For p-tau and the p-tau/Aβ_42_ ratio, CSF P-tau and Aβ_42_ were quantified using commercially available sandwich ELISAs (INNOTEST β-amyloid1–42, and Phospho-Tau[181 P], respectively; Fujirebio Europe, Ghent, Belgium). YKL-40 was quantified using sandwich ELISAs (R&D Systems, Minneapolis, Minn., USA). CSF assays were performed in two batches and corrected for batch differences as previously described^69,70^. For correlational statistical analysis, we used the “cor.test” function in R to calculate Spearman’s rank correlation coefficients between CSF biomarker levels and normalized relative abundances for the 13 differentially abundant genera. The correlation matrix was plotted as ellipses using the *ellipse* package in R.

### Data Availability

The datasets used and/or analyzed during the current study are available from the corresponding author on reasonable request.

## Electronic supplementary material

Supplementary Information
