# Supplementary material for: Gut microbiome alterations in Alzheimer’s disease
Source: Sci Rep. 2017 Oct 19;7:13537. doi: 10.1038/s41598-017-13601-y (PMC5648830; doi:10.1038/s41598-017-13601-y)
Supplement: Supplementary file 1 — Supplementary Information [file 41598_2017_13601_MOESM1_ESM.pdf]

Gut microbiome alterations in Alzheimer's disease

Nicholas M. Vogt[a], Robert L. Kerby[b], Kimberly A. Dill-McFarland[b], Sandra J. Harding[a], Andrew P. Merluzzi[a], Sterling C. Johnson[c,a,d], Cynthia M. Carlsson[c,a,d], Sanjay Asthana[c,a], Henrik Zetterberg[e,f,g,h], Kaj Blennow[e,f], Barbara B. Bendlin[a,d,*], and Federico E. Rey[b,*]

[a]Wisconsin Alzheimer's Disease Research Center, University of Wisconsin School of Medicine and Public Health, 600 Highland Avenue J5/1 Mezzanine, Madison, WI 53792

[b]Department of Bacteriology, University of Wisconsin-Madison, 1550 Linden Drive, Madison, WI 53706

[c]Geriatric Research Education and Clinical Center, William S. Middleton Memorial Veterans Hospital, 2500 Overlook Terrace, Madison, WI 53705

[d]Wisconsin Alzheimer's Institute, University of Wisconsin School of Medicine and Public Health, WARF Building, 610 Walnut Street, 9th Floor, Suite 957, Madison, WI 53726

[e]Institute of Neuroscience and Physiology, Department of Psychiatry and Neurochemistry, The Sahlgrenska Academy at the University of Gothenburg, Mölndal, Sweden

[f]Clinical Neurochemistry Laboratory, Sahlgrenska University Hospital, Mölndal, Sweden

[g]Department of Molecular Neuroscience, University College London Institute of Neurology, Queen Square, London, United Kingdom

[h]UK Dementia Research Institute at University College London, London, United Kingdom

*Co-corresponding authors:
Barbara Bendlin (bbb@medicine.wisc.edu)
Federico Rey (ferey@wisc.edu)

Supplementary Information

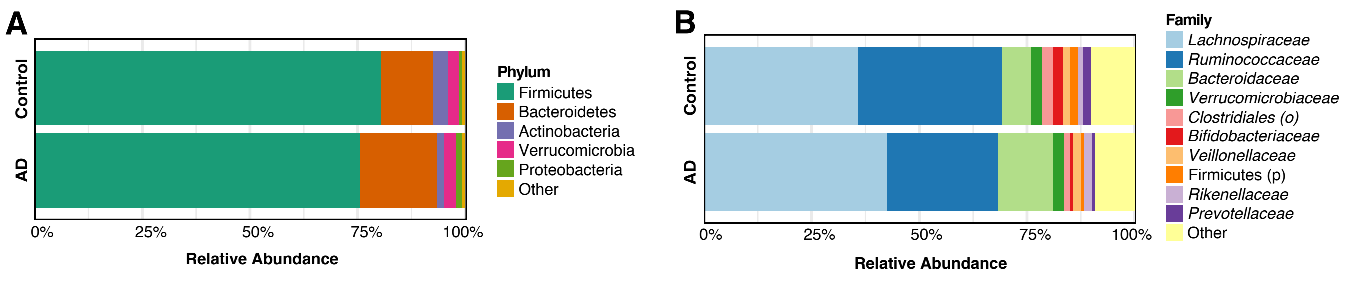

Supplementary Figure S1. Stacked bar plots of average relative abundance at the **(A)** phylum and **(B)** family taxonomic levels. Top 5 phyla (representing 99.3% of all sequence reads) and top 10 families (representing 90.3% of all sequence reads) are shown.

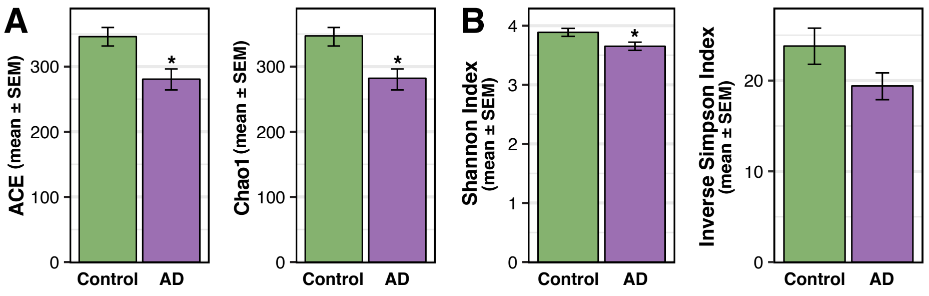

Supplementary Figure S2. The microbiome of AD participants has decreased **(A)** richness (ACE, Chao1) and **(B)** diversity (Shannon Index and Inverse Simpson Index) compared to Control participants. The Abundance-based Coverage Estimator (ACE) and Chao1 provide conservative estimates of microbial richness (# of unique OTUs in a sample). The Shannon Index and Inverse Simpson Index are traditional ecological diversity metrics that take into account richness and abundance of OTUs for each participant. *p<0.05.

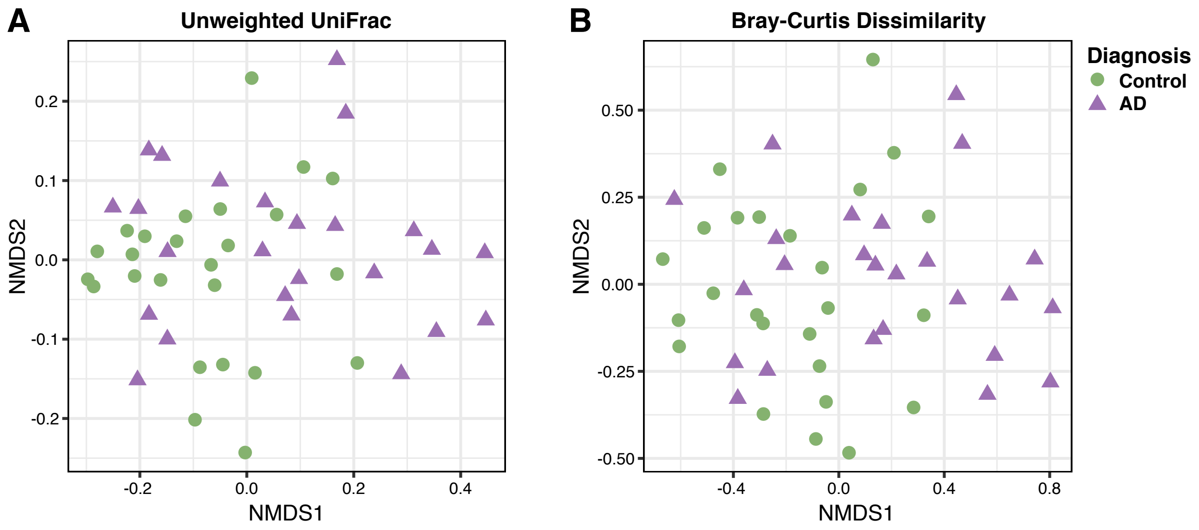

Supplementary Figure S3. Non-metric multidimensional scaling (NMDS) plots of **(A)** unweighted UniFrac and **(B)** Bray-Curtis dissimilarity analyses of relative sample OTU composition. NMDS analysis was limited to two dimensions, with stress measurements of 0.13 and 0.15 for unweighted UniFrac and Bray-Curtis, respectively. Each dot represents a scaled measure of the composition of a given sample, color- and shape-coded by cohort.

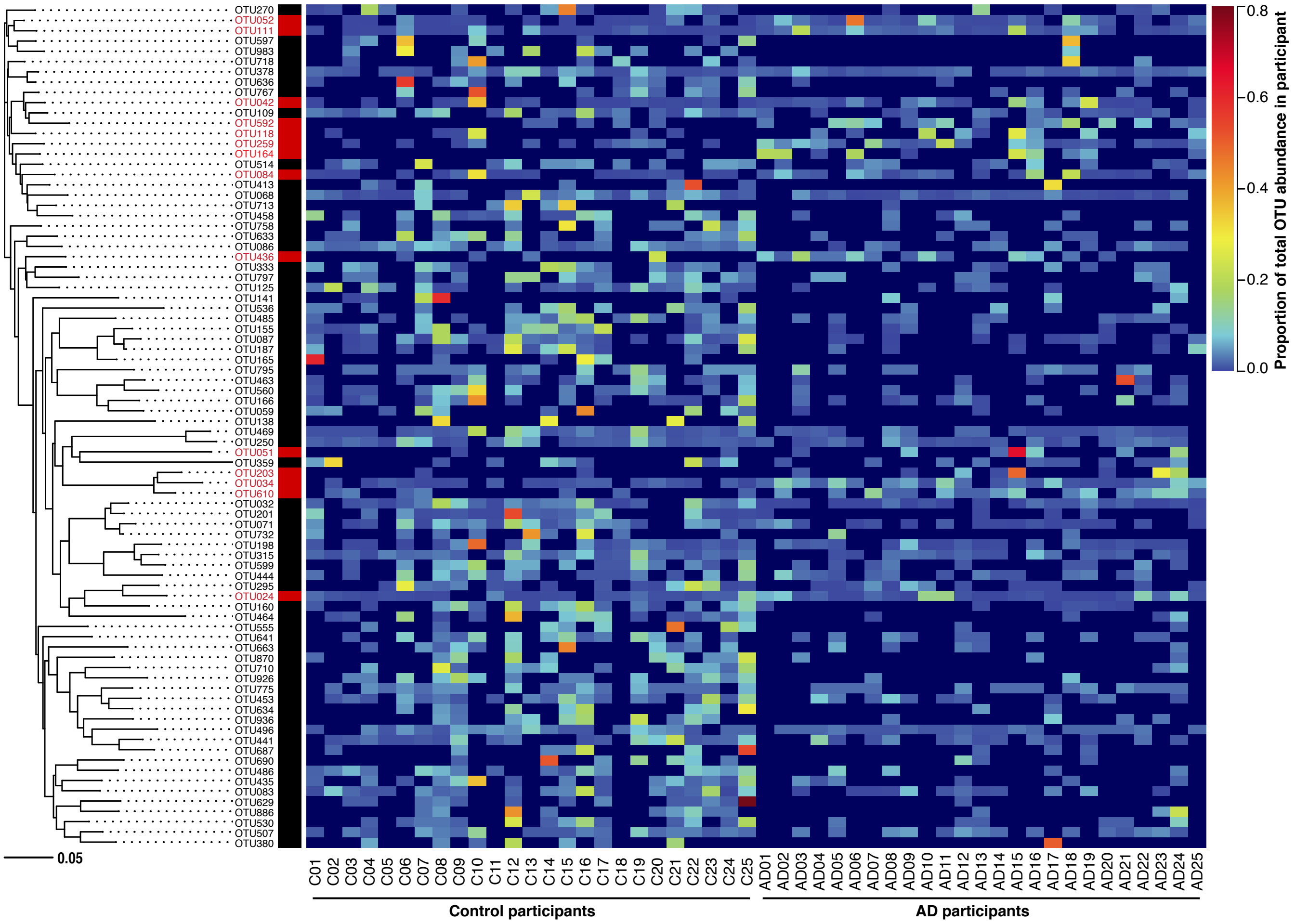

Supplementary Figure S4. Heat map of 82 OTUs differentially represented in AD participants. On left, 16S rRNA sequence-based neighbor-joined tree showing phylogenetic relationships of 82 OTUs identified as differentially abundant in AD participants. Red labels represent OTUs that were increased and black labels represent OTUs that were decreased in AD participants. Data shown for all 50 participants. Color intensities represent relative ratio of OTU abundance in a participant relative to total OTU abundance across all participants.

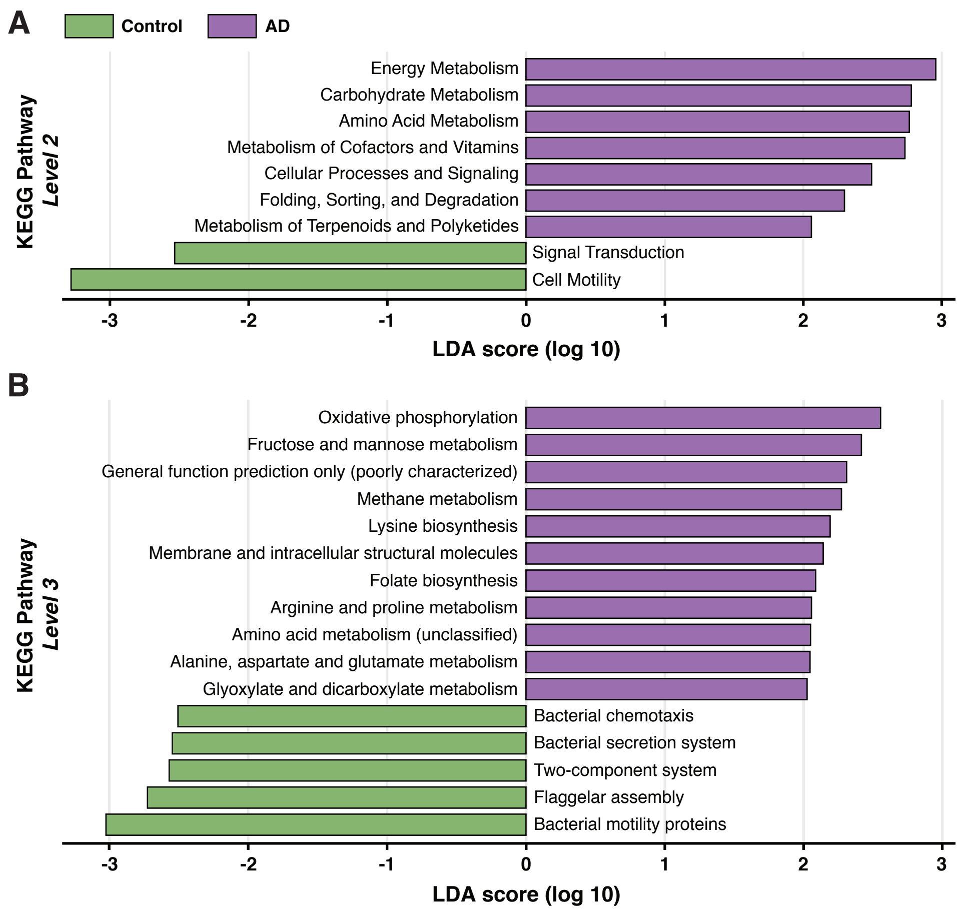

Supplementary Figure S5. Predicted KEGG functional pathway differences at level 2 and 3 between AD (purple) and Control (green) participants. PICRUSt was used to predict functional potential of microbiomes using 16S rRNA gene sequence data. Differentially enriched bacterial functions between groups were identified using LEfSe. Pathway differences plotted as linear discriminant analysis (LDA) scores (log 10). Bars to the right of zero represent bacterial functions enriched in the microbiome of AD participants, while bars to the left of zero represent bacterial functions enriched in the microbiome of Control participants.

Supplementary Figure S6. Correlations between genera relative abundance and CSF levels of YKL-40, a marker of macrophage and microglial differentiation and activation. Correlations were calculated separately for all 40 participants (All), 31 non-demented participants (ND), and 9 AD participants (AD). In AD participants, abundance of *Bacteroides* was positively correlated with CSF YKL-40 levels, while abundance of *Turicibacter* and *SMB53* were negatively correlated with CSF YKL-40 levels. Genera are ordered from most to least abundant. Shape and color of ellipses represent strength of Spearman's rank correlation coefficients (rho). Bolded ellipse borders represent significant correlations (two-sided, *p*<0.05 uncorrected).

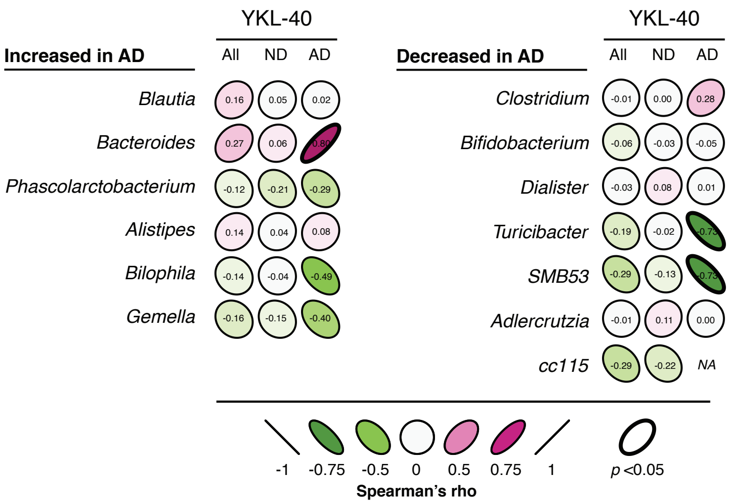

Supplementary Table S1. Medication use information for Control and AD participants.

Medication use	Control	AD	*p* value
Alzheimer's disease medication			
Donepezil/rivastigmine	0/25	23/25	< 0.0001*
Memantine	0/25	14/25	< 0.0001*
Hypertension medication			
ACE-inhibitor/ARB	7/25	7/25	1.000
Beta-blocker	1/25	2/25	1.000
Calcium channel blocker	1/25	3/25	0.609
Diuretic (thiazide)	1/25	2/25	1.000
Statins	7/25	15/25	0.045*
Aspirin	13/25	13/25	1.000
Selective serotonin reuptake inhibitors	1/25	12/25	0.0008*
Atypical antidepressants	4/25	7/25	0.496
Levothyroxine	5/25	6/25	1.000
Proton-pump inhibitors	1/25	7/25	0.0488*
Metformin	2/25	0/25	0.490
Gabapentin	2/25	2/25	1.000
Anxiolytic (benzodiazepine)	2/25	3/25	1.000
Antihistamine (2nd generation)	1/25	3/25	0.609
Anticholinergic	1/25	1/25	1.000
Alendronate	2/25	1/25	1.000

Supplementary Table S2. OTUs differentially represented in feces of AD participants compared to Control participants.

OTU ID	Phylum	Lowest classification	Base Mean	Log_2 Fold Change (±SE)	*p* value (FDR-corrected)
OTU164	Firmicutes	*Dorea*	27.4	6.9 (1.4)	6.78e-05
OTU051	Verrucomicrobia	*Akkermansia*	122.5	5.2 (1.7)	2.31e-02
OTU203	Bacteroidetes	*Bacteroides*	12.9	4.9 (1.8)	4.39e-02
OTU118	Firmicutes	*Blautia*	88.8	4.7 (1.5)	1.84e-02
OTU042	Firmicutes	*Blautia*	201.0	2.9 (0.8)	8.71e-03
OTU436	Firmicutes	*Coprococcus*	1.9	2.9 (1.0)	3.08e-02
OTU052	Firmicutes	*Lachnospiraceae (f)*	323.1	2.8 (0.8)	1.41e-02
OTU592	Firmicutes	*Blautia*	2.6	2.6 (0.7)	4.09e-03
OTU610	Bacteroidetes	*Bacteroides*	5.2	2.4 (0.8)	1.74e-02
OTU259	Firmicutes	*Clostridium*	17.2	2.2 (0.7)	1.89e-02
OTU084	Firmicutes	*[Ruminococcus]*	113.1	2.2 (0.8)	3.96e-02
OTU111	Firmicutes	*Clostridium*	65.3	2.1 (0.5)	8.02e-04
OTU034	Bacteroidetes	*Bacteroides*	537.9	2.0 (0.7)	2.91e-02
OTU024	Firmicutes	*Streptococcus*	432.2	1.8 (0.6)	1.89e-02
OTU138	Firmicutes	*Dialister*	232.2	-8.1 (2.2)	4.56e-03
OTU059	Firmicutes	Firmicutes (p)	505.8	-7.2 (1.5)	5.95e-05
OTU201	Firmicutes	*SMB53*	95.2	-6.8 (1.1)	1.35e-07
OTU141	Bacteria (k)	Bacteria (k)	25.3	-6.7 (2.1)	1.74e-02
OTU160	Firmicutes	*Turicibacter*	173.9	-6.3 (1.1)	1.73e-06
OTU597	Firmicutes	*Dorea*	6.6	-6.2 (1.7)	4.78e-03
OTU165	Firmicutes	*Clostridium*	12.3	-5.9 (2.2)	4.77e-02
OTU270	Firmicutes	*Lachnospiraceae (f)*	65.5	-5.7 (1.2)	1.46e-04
OTU636	Firmicutes	*Lachnospiraceae (f)*	7.9	-5.5 (1.1)	1.97e-05
OTU713	Firmicutes	*Lachnospiraceae (f)*	7.7	-5.5 (1.9)	2.93e-02
OTU087	Firmicutes	*Clostridium*	397.4	-5.2 (1.0)	1.23e-05
OTU413	Firmicutes	*Lachnospiraceae (f)*	3.4	-5.1 (1.9)	4.89e-02
OTU555	Firmicutes	Clostridiales (o)	5.1	-5.1 (1.4)	6.68e-03
OTU155	Firmicutes	*Clostridium*	112.0	-5.1 (1.0)	6.78e-05
OTU380	Firmicutes	*Ruminococcaceae (f)*	2.0	-4.8 (1.6)	2.53e-02
OTU732	Firmicutes	Clostridiales (o)	2.4	-4.7 (1.0)	6.78e-05
OTU634	Firmicutes	*Ruminococcus*	4.0	-4.6 (1.3)	1.04e-02
OTU710	Firmicutes	*Ruminococcaceae (f)*	4.8	-4.6 (1.2)	4.83e-03
OTU633	Firmicutes	*Coprococcus*	6.2	-4.5 (1.0)	1.93e-04
OTU687	Firmicutes	*Ruminococcaceae (f)*	6.8	-4.5 (1.6)	4.34e-02
OTU926	Firmicutes	*Ruminococcaceae (f)*	2.3	-4.4 (0.9)	6.34e-05
OTU463	Firmicutes	Firmicutes (p)	6.8	-4.4 (0.9)	1.45e-04
OTU870	Bacteria (k)	Bacteria (k)	4.1	-4.2 (1.2)	1.04e-02
OTU458	Firmicutes	*Lachnospiraceae (f)*	17.6	-4.1 (1.1)	4.75e-03
OTU071	Firmicutes	Clostridiales (o)	307.8	-4.0 (0.7)	8.19e-06
OTU886	Firmicutes	*Oscillospira*	2.4	-4.0 (1.2)	1.74e-02
OTU718	Firmicutes	*Lachnospiraceae (f)*	2.2	-3.9 (1.4)	3.60e-02
OTU690	Firmicutes	*Oscillospira*	2.3	-3.9 (1.4)	4.86e-02
OTU767	Firmicutes	*Blautia*	4.6	-3.9 (0.9)	7.60e-04
OTU983	Firmicutes	*Lachnospiraceae (f)*	1.8	-3.8 (0.9)	2.51e-04
OTU435	Firmicutes	*Ruminococcaceae (f)*	8.6	-3.8 (1.0)	2.02e-03
OTU032	Firmicutes	*SMB53*	1015.5	-3.7 (0.8)	5.98e-05
OTU629	Firmicutes	*Oscillospira*	1.7	-3.7 (1.0)	2.96e-03
OTU187	Firmicutes	*Clostridium*	68.6	-3.5 (1.1)	1.74e-02
OTU464	Firmicutes	*c115*	22.6	-3.5 (1.2)	3.42e-02
OTU560	Firmicutes	Firmicutes (p)	8.1	-3.5 (0.9)	1.98e-03
OTU333	Firmicutes	*Lachnospiraceae (f)*	34.3	-3.4 (1.1)	2.88e-02

OTU599	Firmicutes	*[Mogibacteriaceae] (f)*	6.8	-3.3 (0.8)	6.44e-04
OTU166	Firmicutes	Firmicutes (p)	113.6	-3.3 (1.1)	2.49e-02
OTU109	Firmicutes	*Blautia*	206.7	-3.3 (0.9)	3.18e-03
OTU663	Firmicutes	*Ruminococcaceae (f)*	5.5	-3.3 (1.1)	2.45e-02
OTU536	Bacteria (k)	Bacteria (k)	2.8	-3.2 (0.9)	1.04e-02
OTU083	Firmicutes	*Ruminococcaceae (f)*	347.6	-3.2 (1.0)	1.84e-02
OTU359	Proteobacteria	*Haemophilus*	10.5	-3.1 (0.9)	1.26e-02
OTU485	Firmicutes	*Christensenellaceae (f)*	10.7	-3.1 (0.8)	3.90e-03
OTU936	Firmicutes	*Ruminococcaceae (f)*	3.0	-3.1 (1.1)	3.72e-02
OTU486	Firmicutes	*Ruminococcaceae (f)*	10.9	-3.1 (1.1)	3.67e-02
OTU758	Firmicutes	*Lachnospiraceae (f)*	2.5	-2.9 (1.0)	2.60e-02
OTU295	Firmicutes	*Lactococcus*	40.6	-2.9 (0.9)	2.07e-02
OTU530	Firmicutes	*Oscillospira*	6.9	-2.8 (1.0)	4.47e-02
OTU125	Firmicutes	*Lachnospira*	107.0	-2.8 (1.0)	4.96e-02
OTU641	Firmicutes	*Ruminococcaceae (f)*	4.4	-2.7 (0.7)	6.28e-03
OTU198	Firmicutes	*[Mogibacteriaceae] (f)*	42.0	-2.5 (0.7)	1.00e-02
OTU086	Firmicutes	*Coprococcus*	225.5	-2.4 (0.9)	4.39e-02
OTU514	Firmicutes	*Lachnospiraceae (f)*	7.1	-2.4 (0.8)	3.42e-02
OTU797	Firmicutes	*Lachnospiraceae (f)*	2.6	-2.3 (0.8)	4.28e-02
OTU441	Firmicutes	*Ruminococcaceae (f)*	14.6	-2.2 (0.8)	3.62e-02
OTU453	Firmicutes	*Ruminococcus*	9.4	-2.0 (0.7)	3.68e-02
OTU315	Firmicutes	*[Mogibacteriaceae] (f)*	23.8	-1.9 (0.6)	1.53e-02
OTU068	Firmicutes	*Lachnospiraceae (f)*	373.9	-1.8 (0.7)	4.89e-02
OTU444	Firmicutes	Clostridiales (o)	7.5	-1.8 (0.6)	3.96e-02
OTU469	Actinobacteria	*Adlercreutzia*	11.7	-1.7 (0.6)	3.01e-02
OTU775	Firmicutes	*Ruminococcus*	3.7	-1.7 (0.5)	1.74e-02
OTU795	Bacteria (k)	Bacteria (k)	2.7	-1.4 (0.5)	3.96e-02
OTU250	Actinobacteria	*Adlercreutzia*	21.4	-1.4 (0.5)	2.45e-02
OTU496	Firmicutes	*Ruminococcaceae (f)*	7.0	-1.4 (0.4)	1.26e-02
OTU507	Firmicutes	*Ruminococcaceae (f)*	5.6	-1.4 (0.5)	4.77e-02
OTU378	Firmicutes	*Lachnospiraceae (f)*	12.4	-1.1 (0.4)	4.34e-02

Supplementary Table S3. Differential abundance at phylum, family, and genus level.

	**Total sequence reads**		**Median, range (%)**			
**Phylum**	**Control**	**AD**	**Control**	**AD**	***p* value (FDR-corrected)**	**↑ or ↓ in AD**
Bacteroidetes	94,050	139,700	12.0, 0.7-42.3	18.3, 2.3-33.9	0.011	↑
Firmicutes	632,303	593,073	82.0, 53.9-93.7	76.5, 57.0-92.3	0.037	↓
Actinobacteria	26,996	14,469	2.37, 0.37-13.2	1.36, 0.21-6.91	0.049	↓
**Family**	**Control**	**AD**	**Control**	**AD**	***p* value (FDR-corrected)**	**↑ or ↓ in AD**
Bacteroidaceae	52,975	100,536	6.23, 0.52-13.7	11.5, 1.7-23.7	4e-4	↑
Rikenellaceae	8,599	14,302	0.82, 0.01-3.61	1.55, 0.01-4.47	0.038	↑
Gemellaceae	18	51	0, 0-0.01	0, 0-0.03	0.048	↑
Ruminococcaceae	262,710	202,254	34.5, 19.3-50.8	27.3, 4.7-42.6	0.008	↓
Bifidobacteriaceae	18,180	6,221	0.94, 0-12.3	0.25, 0-6.71	0.028	↓
Clostridiaceae	16,148	3,456	1.06, 0-9.15	0.10, 0-4.66	0.002	↓
Mogibacteriaceae	2,683	1,052	0.16, 0.01-2.38	0.09, 0.003-0.59	0.043	↓
Turicibacteraceae	1,773	44	0.06, 0-1.13	0, 0-0.04	1e-4	↓
Peptostreptococcaceae	1,038	40	0.003, 0-2.16	0, 0-0.09	0.015	↓
**Genus**	**Control**	**AD**	**Control**	**AD**	***p* value (FDR-corrected)**	**↑ or ↓ in AD**
Blautia	82,787	119,554	8.01, 1.5-30.8	12.9, 2.02-28.0	0.042	↑
Bacteroides	52,975	100,536	6.23, 0.52-13.7	11.5, 1.65-23.7	5e-4	↑
Alistipes	6,294	11,127	0.62, 0.01-2.97	1.13, 0-4.14	0.032	↑
Phascolarctobacterium	2,823	8,984	0.11, 0-1.31	1.13, 0-3.00	5e-4	↑
Bilophila	781	1,606	0.05, 0-0.26	0.21, 0-0.85	0.018	↑
Gemella	18	51	0, 0-0.01	0, 0-0.03	0.049	↑
Bifidobacterium	18,180	6,221	0.94, 0-12.3	0.25, 0-6.71	0.025	↓
SMB53	9,250	1,527	0.51, 0-5.96	0.02, 0-1.25	0.001	↓
Dialister	8,842	1,701	1.20, 0-2.99	0, 0-1.78	5e-4	↓
Clostridium	6,807	1,755	0.28, 0-3.88	0.003, 0-4.41	0.024	↓
Turicibacter	1,773	44	0.06, 0-1.13	0, 0-0.04	1e-4	↓
Adlercreutzia	478	203	0.04, 0.003-0.24	0.02, 0-0.11	0.017	↓
cc115	454	45	0.01, 0-0.38	0, 0-0.07	1.2e-4	↓

Supplementary Table S4. Participant characteristics, CSF biomarker levels, and genus relative abundance.

	All	Non-demented (ND)	AD
n	40	31	9
Age at fecal sample (yrs, mean ± SD)	69.6 ± 8.3	68.3 ± 8.6	74.0 ± 5.1
Age at CSF collection (yrs, mean ± SD)	65.7 ± 7.8	64.5 ± 8.1	70.0 ± 4.8
Sex (% Female)	73% (29/40)	74% (23/31)	67% (6/9)
Ethnicity (% Caucasian)	98% (39/40)	97% (30/31)	100% (9/9)
BMI (kg/m^2, median [IQR])	26.8 [24.6 – 29.4]	26.6 [24.2 – 30.5]	28.7 [25.7 – 29.1]
**CSF biomarkers**	**All**	**ND**	**AD**
$A\beta_{42}/A\beta_{40}$ (mean ± SD)	0.084 ± 0.020	0.091 ± 0.017	0.059 ± 0.006
P-tau (pg/mL, mean ± SD)	52.5 ± 27.7	44.1 ± 15.4	81.4 ± 40.5
P-tau/$A\beta_{42}$ (mean ± SD)	0.100 ± 0.080	0.068 ± 0.039	0.209 ± 0.089
YKL-40 (ng/mL, mean ± SD)	165.8 ± 71.8	152.2 ± 45.5	212.6 ± 119.1
	**Relative Abundance (%), median [range]**		
**Genus**	**All**	**ND**	**AD**
Blautia	9.98 [1.92-30.8]	8.76 [1.92-30.8]	16.4 [10.1-27.0]
Bacteroides	8.52 [0.56-25.9]	6.41 [0.56-25.9]	15.5 [1.65-21.4]
Phascolarctobacterium	0.46 [0-3.00]	0.32 [0-2.35]	1.60 [0-3.00]
Alistipes	0.55 [0-3.52]	0.54 [0-2.05]	0.93 [0-3.52]
Bilophila	0.07 [0-0.41]	0.07 [0-0.28]	0.17 [0-0.41]
Gemella	0 [0-0.03]	0 [0-0.01]	0 [0-0.03]
Clostridium	0.66 [0.04-5.16]	0.84 [0.04-5.16]	0.36 [0.11-4.72]
Bifidobacterium	0.25 [0-9.29]	0.36 [0-9.29]	0.12 [0-6.71]
Dialister	0.02 [0-3.35]	0.68 [0-3.35]	0 [0-1.26]
Turicibacter	0.003 [0-0.55]	0.01 [0-0.55]	0 [0-0.04]
SMB53	0.003 [0-1.01]	0.006 [0-1.01]	0 [0-0.06]
Adlercreutzia	0.02 [0.003-0.13]	0.02 [0.003-0.13]	0.01 [0.003-0.05]
cc115	0 [0-0.23]	0.003 [0-0.23]	0 [0-0]

Supplementary Table S5. Statistics comparing microbial richness, diversity, and differentially abundant genera between AD participants taking selective serotonin reuptake inhibitors (SSRIs) and AD participants not taking SSRIs.

	SSRI	No SSRI	*p* value
n	12	13	--
**Richness**			
ACE (mean ± SD)	287 ± 75	275 ± 87	0.72
Chao1 (mean ± SD)	289 ± 75	275 ± 87	0.67
**Diversity**			
Shannon Index (mean ± SD)	3.68 ± 0.34	3.63 ± 0.36	0.72
Inverse Simpson Index (mean ± SD)	19.8 ± 6.7	19.1 ± 8.2	0.82
Faith's Phylogenetic Diversity (mean ± SD)	10.6 ± 2.5	10.1 ± 2.9	0.70
	**Relative abundance (%), median [range]**		
**Genus**	**SSRI**	**No SSRI**	***p* value**
Blautia	13.4 [6.1-27.0]	12.9 [2.0-28.0]	0.65
Bacteroides	13.2 [1.6-20.2]	9.5 [2.7-23.7]	0.98
Phascolarctobacterium	1.02 [0-2.46]	1.30 [0-3.00]	0.21
Alistipes	1.15 [0.01-3.69]	0.96[0-4.14]	1.00
Bilophila	0.15 [0-0.41]	0.25 [0-0.85]	0.10
Gemella	0 [0-0.2]	0.003 [0-0.03]	0.65
Clostridium	0.01 [0-4.41]	0.003 [0-0.60]	0.57
Bifidobacterium	0.02 [0-1.55]	0.45 [0-6.71]	0.09
Dialister	0 [0-1.26]	0 [0-1.78]	0.47
Turicibacter	0 [0-0.02]	0 [0-0.04]	0.11
SMB53	0.01 [0-0.83]	0.08 [0-1.25]	0.25
Adlercreutzia	0.03 [0-0.11]	0.02 [0-0.05]	0.25
cc115	0 [0-0.05]	0 [0-0.07]	0.77
